# How do scoliotic spines with low stiffness and viscoelastic properties react to the application of spinal orthoses?

**DOI:** 10.1186/1748-7161-10-S1-O61

**Published:** 2015-01-19

**Authors:** MS Wong, M Li, K Luk, K Cheung

**Affiliations:** 1 The Hong Kong Polytechnic University, Hong Kong; 2 The University of Hong Kong, Hong Kong

## Objective

In the management of moderate adolescent idiopathic scoliosis (AIS), spinal orthosis is generally applied. Nonetheless, the time to reach its maximal corrective effect after donning or the time to return to the original scoliotic curvature after doffing may not be well understood because the spine has low stiffness and viscoelastic properties and continuous radiographic assessments are invasive to patients. This study aims to investigate the time dependent response of scoliotic curvature to spinal orthosis by using three-dimensional non-invasive clinical ultrasound (3-D CUS).

## Material and method

In this prospective study, nine female subjects with AIS and under orthotic treatment after adaptation period were recruited. Before joining this study, all subjects were prescribed to wear their orthoses 23 hours a day with the strap tightness determined by an experienced orthotist. It was a two-group (i.e. don-orthosis and doff-orthosis groups) study protocol. The don-orthosis group was designed to investigate the donning effect, while the doff-orthosis group focused on the doffing effect. In the don-orthosis group, 3-D CUS was applied to monitor the spinal curvature changes from 23-hour doff-orthosis stage to immediate don-orthosis and up to 180-minute don-orthosis stage with an interval of 30 minutes. In the doff-orthosis group, CUS was used to evaluate the spinal curvature changes from 23-hour don-orthosis stage to immediate doff-orthosis and up to 180-minute doff-orthosis stage with an interval of 30 minutes. The spinous process angle (measured from the ultrasound image), which was used to estimate the Cobb angle (generally measured from radiograph), was employed as an indicator to assess the changes of scoliotic curvature.

## Results

The changes of scoliotic curvature (Cobb angle) at the stages of immediate don-orthosis and doff-orthosis were not obvious. In the monitoring process, the curvatures decreased > 5º at or after 30-minute don-orthosis and increased > 5º at or after 30-minute doff-orthosis. The duration varied in different individuals. After 120 minutes don-orthosis or doff-orthosis, all curves tended to be steady.

## Conclusion

This investigation demonstrated that the response of scoliotic spine on orthotic intervention was lagged owing to its low stiffness and viscoelastic properties. The best correction happened 120 minutes after donning the spinal orthosis and the correction could not be maintained at and after 120 minutes of doffing the spinal orthosis. Further studies with larger sample size should be warranted to confirm the current observation and facilitate comprehensive understanding the mechanism of orthotic intervention to the patients with AIS.

